# Parents’ experiences caring for children with acute otitis media: a qualitative analysis

**DOI:** 10.1186/s12875-022-01737-4

**Published:** 2022-05-23

**Authors:** Matthew C. Lee, Dio Kavalieratos, Anastasia Alberty, Destin Groff, Mary Ann Haralam, Nader Shaikh

**Affiliations:** 1 Division of General Academic Pediatrics, Children’s Hospital of Pittsburgh of UPMC, University of Pittsburgh School of Medicine, Pittsburgh, PA USA; 2 Department of Family and Preventive Medicine, Emory University School of Medicine, Atlanta, GA USA

**Keywords:** Acute Otitis Media, Parent Perspectives, Qualitative Interviews, Symptom severity

## Abstract

**Background:**

Little is known regarding parents’ experiences caring for children with acute otitis media (AOM). This study aimed to explore parents’ experiences caring for their child with AOM, identifying symptoms they observed, their thoughts and feelings about those symptoms, how they managed the episode, and what factors caused them to seek medical evaluation.

**Methods:**

From October 2019 to February 2020, we conducted 24 semi-structured cross-sectional interviews with parents of children 3 to 36 months of age with AOM diagnosed at primary care offices associated with the Children’s Hospital of Pittsburgh regarding (1) symptoms and behaviors that led parents to believe their child might have AOM; (2) symptoms that were most bothersome to parent and child; (3) what parents did in response to these symptoms; (4) motivations for seeking clinical care; and (5) parents’ expectations regarding AOM resolution. Data were analyzed using template analysis, resulting in a hybrid inductive/deductive analytic process.

**Results:**

We interviewed 24 parents within 72 h of diagnosis of AOM. Parents frequently believed ear tugging was the symptom most indicative of AOM, despite its presence in only half of the children in this sample. Parents consistently sought medical care when their child had an elevated temperature or lack of sleep, or when symptoms worsened or were unresponsive to home remedies. Parents of children with history of recurrent AOM had less difficulty identifying symptoms of AOM than parents of children with their first ear infection.

**Conclusions:**

Our findings provide insight into symptoms of AOM that cause parents concern and motivate the use of healthcare services. Parents differed in their abilities to observe and report symptoms of AOM. Thus, when interviewing parents who are concerned their preverbal child has AOM, rather than focusing on ear tugging and fever alone, providers should ascertain all unusual behaviors observed by the parent.

## Key points

*Question*: What behaviors and symptoms do parents of children with ear infection notice, which ones do they find most concerning, and what actions do they take to address these concerns?

*Findings*: Parents noticed many abnormal behaviors in their children. Many symptoms led to parental distress. Symptoms that caused concern frequently caused parents to seek medical care. 

*Meaning*: Parents differed in their abilities to observe and report symptoms of AOM. Thus, when interviewing parents who are concerned their preverbal child has AOM, rather than focusing on ear tugging and fever alone, providers should ascertain all unusual behaviors observed by the parent.

## Background

Except for the common cold, acute otitis media (AOM) is the most frequently diagnosed illness in children in the United States [1]. By age three, 80% of children will have at least one episode of AOM [2]. Furthermore, AOM is the leading cause of antimicrobial prescription in preverbal children [3].

While other qualitative studies have reviewed parents’ beliefs about AOM treatment and management [4–6], parent’s experiences consulting for AOM [7], and parents’ gaps in knowledge of AOM [8], few qualitative studies have focused on signs and symptoms parents noticed in their preverbal child, and how parents respond to those signs and symptoms. We sought to investigate parents’ experiences caring for children 3–36 months with recently diagnosed AOM, exploring symptoms and behaviors parents notice when their child becomes ill, their thoughts and feelings about those symptoms, and symptoms that influenced them to seek medical evaluation.

## Methods

### Design

We conducted a phenomenological qualitative study to understand the experiences of parents caring for a child recently diagnosed with AOM. We chose semi-structured cross-sectional interviews as our key data collection method, as the flexible nature of this interviewing style allows for exploration of topics discussed by the participant, while maintaining overall consistency across interviews. To reduce burden on parents, interviews were conducted via telephone. Interviews took place within 72 h of diagnosis, when most children with AOM were still symptomatic. The University of Pittsburgh Institutional Review Board approved this study.

### Sample and recruitment

We recruited parents of children, ages 3 to 36 months, with clinically diagnosed AOM presenting to any of 4 outpatient clinics, 1 urban and 3 suburban, affiliated with the UPMC Children’s Hospital of Pittsburgh. We used stratified sampling across 3 age ranges: 3–12, 12–24, and 24–36 months. We included only children with AOM defined by the presence of moderate or marked tympanic membrane bulging, or slight bulging accompanied by marked tympanic membrane erythema. We excluded children with perforated tympanic membranes or tympanostomy tubes, as they may present with different symptoms. We excluded children with evidence of another presumed bacterial infection, children with an underlying immune deficiency, and children whose parents were unable to read or write in English. Verbal informed consent was obtained from all participating parents.

### Data collection

Interviewers followed an interview guide (Table 1) created by a qualitative researcher (DK) and revised per input from the research team. The guide contained nine core questions that were relevant and appropriate to the domains our study sought to address: the child’s symptoms, the parent’s concerns, and the parent’s actions related to the child’s symptoms. The guide was pilot tested prior to use in this study. Interviews were conducted by AA, DG and NS over the phone from October 2019 to February 2020. Phone calls were recorded with patient consent. Only one researcher and participant were present during each interview. Interviews were transcribed verbatim using a professional transcription service, with text files compared to audio recordings to ensure transcription accuracy. We concluded enrollment in the study when we reached thematic saturation (i.e., the point at which the incremental yield from each additional interview is insufficient to justify further data collection).

**Table 1:** Interview guide

**1.** Let’s get started with a bit of a big picture question to help me learn about you and [child]. In your own words, please tell me everything that led up to [child] being diagnosed with an ear infection.
**2. **What led you to think that [child] might have some ear troubles?
**3.** Sometimes children do specific things that we think might tell us they are having ear trouble. **a.** [For each sign/symptom mentioned]: What behaviors were you noticing [child] doing? **b.** How did you first notice that [child] was having [symptom]? **c.** If the child is >1 year old, ask “what words did your child use to communicate the [symptom]? **d.** What did you think was causing the [symptom]? **e.** What was it like for you to see [child] do [behavior]?
**4.** Of all the symptoms you mentioned, which ones do you think were the most bothersome to [child]? **a.** Which of those symptoms were the most bothersome to *you*?
**5.** Once you noticed [child] your child’s symptoms, what did you do? **a.** How could you tell that the symptoms were getting worse?
**6.** Tell me about how you decided you needed to seek help. What was the thing that pushed you to call your pediatrician?
**7.** Did you think your child was in pain? If so, how could you tell?
**8.** Can you spend some time telling me about how you’d know whether [child]’s ear troubles are fixed? What would that look like? **a.** Some parents say that they feel confident that their child is better when they do certain things or stop doing other things.
**9.** Now that we are wrapping up, is there anything else you would like to share with me or that you think is important for us to know?

We undertook several steps to ensure research rigor [9]. First, DK trained interviewers in qualitative interviewing skills using role plays to practice key interviewing techniques. Second, DK audited every third transcript during data collection to provide interviewers with feedback, and to monitor progress toward thematic saturation. Third, we held weekly debriefing meetings to discuss interview quality.

### Analysis

We analyzed data using template analysis, a qualitative approach that combines content analysis and grounded theory, resulting in a hybrid inductive/deductive analytic process [10]. AA developed the initial codebook based on themes from a sample of 4 interviews. The codebook was approved by DK and NS. Using the initial codebook, the transcripts were coded in a deductive fashion by ML. After every four interviews, coding was reviewed by DK to ensure agreement among the study team. Coding disagreements were resolved by discussion until there was full consensus. As more transcripts were coded, the codebook was refined to more accurately capture themes emerging from the interviews. NVivo 12 Pro software was used to code and query transcripts. Code trees and other cluster maps generated with NVivo were used to explore emerging themes.

## Results

Of the 45 participants verbally consented at the time of AOM diagnosis, 24 (53%) completed interviews by phone within 72 h of diagnosis; 21 participants could not be reached within the study required 72-h window after having consented to the study. These individuals did not differ in age, sex, or race from parents who participated (data not shown). The mean interview length was 13 min and 38 s.

Our sample was racially and socioeconomically diverse (Table 2). A total of 21 (88%) of parents interviewed were female. Of note, 7 (29%) children were verbal to some degree (Table 2) and 4 (17%) were able to verbally communicate their ear pain. Seven qualitative themes with sub themes are presented below and summarized in Table 3.

**Table 2:** Characteristics of study sample

Characteristic		Full Sample	< 12mo	12-24mo	> 24mo
		*n* = 24	*n* = 10	*n* = 10	*n* = 4
Age (months)	Mean	16.0	8.1	18.4	29.25
	Range	6–32	6–11	13–21	27–32
Race					
	White	13	7	5	1
	Black	11	3	5	3
Child Sex					
	Male	15	5	6	4
	Female	9	5	4	0
Parent Sex					
	Male	3	2	1	0
	Female	21	8	9	4
Health Insurance					
	Public	13	3	7	3
	Private	11	7	3	1
Verbal Child					
	Verbal	7	0	3	4
	Non-Verbal	17	10	7	0

**Table 3:** Themes identified

Theme	Sub Theme	Text
Identifying AOM	Most parents looked for ear tugging to identify AOM	“when I noticed she was pulling her ears, I was going to take her to the emergency room, but I knew she had a doctor's appointment today.” (21)
	In absence of otalgia, parents attributed other symptoms to diseases other than AOM	“I didn't know if it was teething, just the fever. I didn't know. Because he didn't grab his ears or anything” (11)
	Verbalized otalgia made parents more confident in their assumption of AOM	“he's telling me it hurts, so now I can, ‘Oh, okay, we need to go to the doctor's. You've got another ear infection.’” (32)
Experience caring for a child with AOM	Parents of children with history of AOM suspected AOM often	“he started not sleeping, so then I was like, ‘Ah I bet it's his ears.’” (15)
	Parents of children with no history of AOM generally did not suspect AOM if ear tugging was absent	“I actually didn't think that it was an ear trouble. She hasn't been suffering [inaudible] or anything, it's just that she wasn't sleeping.” (24)
Symptoms that bothered child vs. bothered parent	Symptoms that most troubled parents were often reported as the same symptom that most bothered their child	“Which [symptoms] were most bothersome to you?” (interviewer) “I think the fever too, just because it was hard to manage.” (11)
Behaviors that led to parental distress	Parents reported feelings of helplessness and sadness when children were difficult to console	“Awful. Completely awful. I feel helpless.” (22)
Parents’ efforts to console their child	Parents devoted more attention to their children	“I pick her up. I cradle her, rock her back and forth in my arms. I try to rub her back, give her kisses.” (26)
	Parents administered analgesics, antipyretics, and home remedies to their ill children	“I was using a warm compress… to kind of calm or kind of keep him a little bit happier” (43)
Factors associated with the decision to seek medical evaluation	Parents sought medical care when they believed they could not manage their child’s symptoms without assistance	“the fact that I can't really calm him down and comfort him showed that I needed to get more help than just me” (22)
	Fever and disturbed sleep were symptoms that caused parents concern and instilled a sense of urgency to take their child to a physician	“I can take care of that, like a cough, sore throat, stuff like that. But the fever is what would really scare me” (18) “[He] just was still not sleeping and just being fussy during the day and it just– I wasn't sleeping, and so that's when I was like, ‘I guess I'm just going to have him go checked out’” (15)
How parents determine when AOM has resolved	Parents stated that their child being back to their ‘normal self’ signified AOM resolution	“Just back to his normal routine. That usually tells me that he's better.” (11)

### Identifying AOM

Symptoms parents often reported included crying, fussiness, sleep disturbance, fever, respiratory and nasal symptoms, decreased oral intake, unaccustomed ear rubbing or tugging (hereinafter referred to as ear tugging) and decreased activity.

Approximately half of parents reported their child displayed ear tugging. Almost all parents who observed ear tugging suspected AOM and cited this behavior as the reason for seeking medical evaluation.

*Participant 21- Mother of a 6-month-old*

21: So yesterday, when I noticed she was pulling her ears, I was going to take her to the emergency room, but I knew she had a doctor's appointment today. So that's why I didn't follow-up.

Interviewer: Okay. And so, if you did not have this appointment today, what would have prompted you to go to the doctor's office?

21: Her pulling on her ears

When ear tugging was absent, parents often suspected other acute illnesses as the cause of their child’s symptoms and behaviors, such as the common cold, teething and/or the flu.

*Participant 18- Mother of a 20-month-old*

“I thought he had the flu ... He was really sick, but he didn't show the normal signs and symptoms.”

*Participant 11- Mother of a 19-month-old*

“I didn't know if it was teething, just the fever. I didn't know. Because he didn't grab his ears or anything, so I wasn't sure if that was it. But he just looked uncomfortable.”

Many parents reported that their child had pain not specific to the ears; some reported ear pain (hereinafter referred to as otalgia). Parents used observable symptoms to infer that their child was experiencing pain. Overwhelmingly, parents identified crying as the primary reason they inferred pain in their child. Some parents noted crying in combination with other behaviors, such as ear tugging and facial expressions, as indicative of otalgia.

*Participant 30- Mother of a 17-month-old*

“Yes, I definitely thought she was in pain. And the screaming was a pretty strong indicator that she was in a lot of pain, the poor kid”

*Participant 17- Mother of a 9-month-old*

17: Because two nights went by where she was screaming, and then that's when I noticed the ear pulling.

Interviewer: And what was the thing that pushed you to call your pediatrician?

17: Worrying that the ear would get worse and her being in more pain.

Parents seemed more confident in their ability to identify an ear infection when their child verbalized their otalgia.

*Participant 32- Mother of a 31-month-old*

“So now that he talks, he's telling me it hurts, so now I can, ‘Oh, okay, we need to go to the doctor's. You've got another ear infection.’ Now, before, when he cried, I didn't know he had it, and he had it, probably, for days. …. So it's a big difference.”

### Experience caring for a child with AOM

Parents of children with a history of AOM often suspected AOM as the cause of their child’s symptoms, even in the absence of ear tugging. On the whole, these parents exhibited more confidence in their prediction that their child had AOM.

*Participant 15- Mother of a 11-month-old with history of AOM*

“[He] started out with a runny nose, and then got a cough, and in the past he's had several ear infections, and I waited and waited and waited for it to clear up. It didn't clear up. Then he-- I guess two weeks after it began he started not sleeping, so then I was like, ‘Ah I bet it's his ears’… he's had three other ear infections, and that's the only time he doesn't sleep”

In contrast, if a parent had no history of caring for a child with AOM, and the child did not display ear tugging, the uninitiated parent generally did not suspect AOM as the cause of their child’s illness.

*Participant 24- Mother of a 7-month-old with no history of AOM*

“I actually didn't think that it was an ear trouble. She hasn't been suffering [inaudible] or anything, it's just that she wasn't sleeping. And it just seemed like she was bothered.”

Even for parents whose child had a history of AOM, discerning their child had AOM was sometimes difficult when the child’s symptoms were not consistent with their prior episodes of AOM.

*Participant 11- Mother of 19-month-old with history of AOM*

“I thought he could [have ear troubles] because he's had history of ear infections and the cold. Sometimes, like I said, his nose is draining and he gets an ear infection. But he didn't actually grab his ears or pull at his ears or anything. I only found that out whenever they checked them just because of his history.

### Symptoms that bothered child vs. bothered parent

Symptoms that most troubled parents were usually the same symptom they believed most bothered their child. This may have been related to parent’s inability to console their child and/or manage their child’s symptoms.

*Participant 11- Mother of 19-month-old*

Interviewer: which [symptoms] do you think were the most bothersome to your child?

11: I think the fever, because he normally does not get fevers and he can be sick and completely happy. But with the fever this time, he was way more irritable…

Interviewer: And of all the symptoms again, which ones were most bothersome to you?

11: I think the fever too, just because it was hard to manage.

### Behaviors that led to parental distress

When prompted with “what was it like for you to see your child like this?” parents often replied with emotionally loaded words. Feelings of helplessness and sadness were present in multiple interviews, and most of these interviews featured fussy children who were noted to be difficult to console. Emotional distress frequently appeared to be related to the parent’s inability to manage their child’s symptoms, and/or inability to determine the reason for their child’s symptoms.

*Participant 22- Mother of 6-month-old*

Interviewer: And what was it like for you to see your child like this?

22: Awful. Completely awful. I feel helpless.

*Participant 24- Mother of 7-month-old*

“It makes me sad. She can't tell me what's wrong. So you're trying everything to figure out what's wrong. Trying to wipe her tears away. Because you don't want nothing to be wrong with your little baby.”

### Parent’s efforts to console their child

Consoling took many forms, such as increased contact, which included picking up, holding, rubbing, rocking, and/or kissing the child. It also included providing the child comfort items such as blankets, stuffed animals, toys, a bottle and/or a pacifier. Parents appeared to give their child attention to quell the child’s perceived frustration and discomfort. Furthermore, parents reported that these efforts often only worked while the parent actively gave the child attention.

*Participant 26- Father of 10-month-old*

“I pick her up. I cradle her, rock her back and forth in my arms. I try to rub her back, give her kisses. I would try to give her a teddy bear or something that maybe that she likes to hold. Try to give it to her. But it's like she doesn't want any of that stuff, and she's really upset”

Parents typically administered acetaminophen and ibuprofen primarily to reduce their child’s fever, but also to reduce any pain their child may have experienced.

*Participant 13- Mother of a 21-month-old*

“I mean, it was like Wednesday and Thursday was non-stop. She would not stop crying… we would give her the Motrin and it would kind of relieve her.

Parents that believed their child was experiencing AOM also tried home remedies that they believed would relieve otalgia.

*Participant 43- Mother of a 27-month-old*

“I was using a warm compress, just to kind of wring out all the water and everything but then put that on the side of his ears to kind of calm or kind of keep him a little bit happier or maybe relieve some of the pressure if it was.”

### Factors associated with the decision to seek medical evaluation

Parents sought medical care when they felt they could no longer manage their child’s symptoms without assistance.

*Participant 22- Mother of 6-month-old*

“This is my third child, and I've been not new to this, so I mean, the fact that I can't really calm him down and comfort him showed that I needed to get more help than just me”

Additionally, parents who suspected pain sought medical evaluation to prevent their child from experiencing unnecessary pain.

*Participant 17- Mother of 9-month-old*

Interviewer: And what was the thing that pushed you to call your pediatrician?

17: Worrying that the ear would get worse and her being in more pain.

Parents most frequently cited fever as the symptom that motivated them to seek medical care. The urgency with which parents sought medical care seemed to be associated with their level of concern accompanying fever in their child.

*Participant 26- Father of 10-month-old*

“As far a runny nose and fussiness and stuff like that, I'd let that go a couple days usually, but once they get a fever, it's like I'll take them in”

*Participant 18- Mother of 20-month-old*

“It was really the fever that got me because an average cold, I can take care of that, like a cough, sore throat, stuff like that. But the fever is what would really scare me”

Disruption of the child’s sleep also motivated parents to seek medical care. Crying and/or coughing during the night tended to cause parents more concern compared to crying and coughing during the day. Being kept awake at night seemed to mentally tax parents.

*Participant 15- Mother of 11-month-old*

“[He] just was still not sleeping and just being fussy during the day and it just-- I wasn't sleeping, and so that's when I was like, "I guess I'm just going to have him go checked out," … And it just makes for an extremely long day when he doesn't take one single nap and then you're up all night too, so. It is tough.”

### How parents determine when AOM has resolved

Most parents felt that their child being back to their ‘normal self’ was sufficient evidence of AOM resolution. Only a subset of parents mentioned that a clinician’s opinion would make them more confident that the infection had cleared.

P*articipant 11- Mother of 19-month-old*

Interviewer: So could you spend some time telling me about how you would know whether your child's ear troubles are fixed?

11: This time, well, I mean, definitely the fever I'd look for, but also just the follow-up appointment. We come back soon, so just double check. …Just back to his normal routine. That usually tells me that he's better.

## Discussion

In this qualitative study, parents often discussed crying, fussiness, sleep disturbance, fever, respiratory and nasal symptoms, decreased oral intake, ear tugging, and decreased activity as symptoms they saw in their child with AOM. Parents frequently relied on ear tugging to infer if their child had AOM, and this was especially true for parents of children with no history of AOM. In response to these symptoms, parents gave their children increased attention, analgesics/antipyretics, and other home remedies to ease inferred otalgia. In the absence of ear tugging, parents often attributed symptoms to conditions other than AOM. Seeing their child ill often caused parental distress, and feelings of sadness and helplessness. Fever and lack of sleep were symptoms that most concerned parents and were often cited as the reason for seeking medical evaluation. Parents also sought medical care once they felt they could no longer manage their child’s illness alone. Lastly, parents believed that their child returning to their ‘normal self’ was sufficient evidence that AOM had resolved.

Themes identified here are consistent with the existing literature. Symptoms parents reported (i.e., crying, fussiness, sleep disturbance, fever, respiratory symptoms, decreased oral intake, ear tugging, and decreased activity) are largely consistent with previous reports [4–8, 11–14]. Similar to our study, a previous qualitative study that found fever was a symptom that caused parents the most concern [15], and another study that found sleep disturbance to be most burdensome [11]. Parents’ difficulty differentiating AOM from teething or the common cold [6] and parents’ feelings of helplessness, sadness, and anxiety have also been reported in previous studies [5, 7, 8, 11, 15]. Finally, parents’ decision to consult a clinician to prevent their child from experiencing any further pain or because they felt they could no longer manage their child’s symptoms alone has been reported [4, 6, 15, 16].

This study also uncovered new themes not previously discussed in the literature. We found that parents largely rely on ear tugging to infer whether their preverbal child has AOM. While otalgia is an important symptom of AOM, ear tugging is absent in many children with AOM [12]. This may lead to missed cases of AOM. We also found that most parents believed their child’s AOM was successfully treated when their child returned to their ‘normal self.’

Our study had several limitations. First, we interviewed only one parent (often the mother); other caregivers could have had different perspectives. Second, the potential for recall bias cannot be ruled out. However, we completed interviews within 72 h of diagnosis, and this window was more stringent than previous qualitative studies [4, 7, 8]. Our study featured several strengths. First, the use of rigorous qualitative methods ensured we collected quality data, and our analysis was performed properly. Secondly, the exclusion of parents of children with tympanostomy tubes or tympanic membrane perforation ensured we did not see the dilution of typical AOM symptoms, as children with ear drainage tend to present differently and often experience symptom relief. Lastly, the focus on parents of preverbal children was important, as parents of children with the ability to verbalize their otalgia would likely have different experiences from parents of preverbal children. Some previous qualitative studies have not focused their population of parents to parents of preverbal children [6–8].

This study adds to the literature regarding parents’ perspectives while caring for a young child with AOM. Data provided here provides insight into what parents watch for when their child becomes ill, the drivers of their healthcare seeking behavior, and their thoughts and feelings from symptom onset through AOM resolution. Parents differed in their abilities to observe and report symptoms of AOM. Thus, when interviewing parents who are concerned their preverbal child has AOM, rather than focusing on ear tugging and fever alone, providers should ascertain all unusual behaviors observed by the parent.

## Data Availability

The datasets generated and analyzed during this study are not publicly available due to identifying information within the transcripts, but are available from the corresponding author on reasonable request.

## Abbreviation

AOM: Acute Otitis Media
